# Development, characterization, and cross‐amplification of microsatellite markers for *Psammosilene tunicoides* (Caryophyllaceae)

**DOI:** 10.1002/aps3.1199

**Published:** 2018-12-04

**Authors:** Ai‐Li Zhang, Ya‐Fang Gao, Guo‐Dong Li, Zi‐Gang Qian

**Affiliations:** ^1^ Faculty of Traditional Chinese Pharmacy Yunnan University of Traditional Chinese Medicine Kunming Yunnan 650500 People's Republic of China

**Keywords:** Caryophyllaceae, cross‐amplification, genetic diversity, microsatellite, *Psammosilene tunicoides*, *Silene*

## Abstract

**Premise of the Study:**

*Psammosilene tunicoides* (Caryophyllaceae) is a narrowly distributed and endemic plant species in southwestern China. The overexploitation of natural *P. tunicoides* has led to the destruction of many populations. Population and genetic studies will provide crucial data for the protection and management of *P. tunicoides*. In this study, we develop simple sequence repeat markers of *P. tunicoides* to analyze population diversity.

**Methods and Results:**

Microsatellite loci of *P. tunicoides* were isolated with FIASCO. Eleven polymorphic and 10 monomorphic primers were developed. The 11 polymorphic primers were tested in three *P. tunicoides* populations, yielding two to nine alleles per locus. Levels of observed heterozygosity varied from 0.000 to 1.000, and levels of expected heterozygosity ranged from 0.000 to 0.615. In addition, three of these loci were successfully amplified, and showed polymorphism, in three *Silene* species.

**Conclusions:**

These microsatellite markers can be valuable tools to investigate the genetic diversity and population structure of *P. tunicoides*.

*Psammosilene tunicoides* W. C. Wu & C. Y. Wu (Caryophyllaceae), a perennial and monotypic herb endemic to southwestern China, was described more than 500 years ago and is highly valued in traditional Chinese medicine for pain relief, coagulative effects, and promoting blood circulation (Qu et al., 2011). However, population sizes of this species have been declining dramatically in recent years due to overharvesting, and it is currently listed in the *China Plant Red Data Book* as a rare and endangered species (Fu and Chin, 1992). This species urgently requires protection. Previous genetic diversity analysis developed for *P. tunicoides* conservation strategies were mostly dependent on molecular markers, including amplified fragment length polymorphisms (AFLP) (Dai et al., 2007), direct amplification of length polymorphisms (DALP) (Qu et al., 2010; Li et al., 2016), and DNA sequencing (Zhang et al., 2011). The DALP and AFLP markers are often composed of multiple fragments in large genome templates, which complicates their use for genetic analysis.

As molecular markers, microsatellites (also known as simple sequence repeats [SSRs]) are DNA motifs composed of one to six nucleotides, which have gained considerable importance in plant genetics analysis and breeding due to their many desirable attributes, including codominant inheritance, stability, extensive genome coverage, and amenability to automation. SSRs have been found ubiquitously in genetic diversity research, genome evolution, species conservation, and marker‐assisted selection breeding (Cavagnaro et al., 2010; Kalia et al., 2011; Wei et al., 2011; Passos et al., 2013). Because *P. tunicoides* is an endangered species and cultivated herb, it is necessary to develop SSR markers for both conservation strategies and marker‐assisted selection breeding. However, the National Center for Biotechnology Information (NCBI) database contains no SSR sequences for *P. tunicoides* based on Sanger sequencing data. In this study, we report the development and characterization of 11 novel polymorphic genomic SSR markers for *P. tunicoides*. Additionally, we cross‐amplified these loci in three species of the genus *Silene* L.: *S. gracilicaulis* C. L. Tang, *S. huguettiae* Bocquet, and *S. gonosperma* (Rupr.) Bocquet.

## METHODS AND RESULTS

Total genomic DNA was extracted from silica gel–dried leaf tissue from seven samples of *P. tunicoides* from different populations (Appendix 1) using the DNeasy Plant Mini Kit (Tiangen Biotech, Beijing, China). These microsatellite markers were developed using the Fast Isolation by AFLP of Sequences COntaining repeats protocol (FIASCO) with modifications (Zane et al., 2002). Approximately 500 ng of genomic DNA was digested with *Mse*I restriction enzyme (New England Biolabs, Beverly, Massachusetts, USA). Then, using the universal adapter pair (F: TACTCAGGACTCAT, R: GACGATGAGTCCTGAG) combined with its fragment, the digested product was placed at 37°C. This mixture was amplified by PCR using a reaction program containing an initial denaturation of 94°C for 3 min; followed by 20 cycles of 94°C for 30 s, 55°C for 60 s, and 72°C for 60 s; with a final extension of 72°C for 8 min.

PCR product hybridization was performed with 5′‐biotinylated (AC)_15_/(AG)_15_ probes, and hybridization products were enriched using magnetic beads. The collected enriched products were used as a template to conduct a PCR reaction according to the above program. After the amplified product was purified, the purified product was ligated into a pGM‐T vector (Tiangen Biotech) and transformed using Trans1‐T1 Phage Resistant Chemically Competent Cells (TransGen Biotech, Beijing, China) to carry out amplification and expression. Positive strains were selected using Luria–Bertani medium containing ampicillin. A total of 359 positive clones were selected and sequenced using the ABI PRISM 3730XL DNA sequencer (Applied Biosystems, Foster City, California, USA), including 217 sequences containing an SSR. Out of these 217 sequences, 46 successfully yielded clear bands; the others showed multi‐banding patterns or no amplification.

Polymorphisms were validated using the 46 designed primers in 30 samples of *P. tunicoides* that were collected from three locations in China: Yunnan, Sichuan, and Guizhou (Appendix 1). PCR was performed in a 25‐μL reaction volume, containing 1 μL of template DNA (5 ng/μL), 0.5 μL of reference primer, 1 unit of *Taq* polymerase (TaKaRa Biotechnology Co., Dalian, China), 2.5 μL of 10× PCR buffer, 2.0 μL of MgCl_2_ (25 mM), 0.5 μL of dNTP mixture (10 mM each), and 17.8 μL ddH_2_O. The amplification reaction was conducted using the following protocol: initial denaturation at 95°C for 3 min; followed by 30 cycles of 95°C for 30 s, 45–56°C for 1 min (Table 1), and 72°C for 30 s; and a final extension at 72°C for 10 min. After PCR amplification, PCR products were separated and visualized using an ABI 3730 automated sequencer (ABI 3730XL, Applied Biosystems), and the size of the alleles at each locus was scored by GeneMapper version 3.2 (Applied Biosystems). All sequences were deposited in GenBank (Table 1).

**Table 1:** Characteristics of 11 polymorphic microsatellite loci developed for *Psammosilene tunicoides*

Locus	Primer sequences (5′–3′)	Repeat motif	*T*_a_ (°C)	Allele size range (bp)	*A*	GenBank accession no.
E2	F: TCCCTCCATACTCATACA	(GAA)_5_	45	278–284	4	KJ159956
	R: ATGCAAACCTTATTCTTC					
E5	F: TCCGACGAAGGGAATGCT	(GA)_10_	53	175–179	3	KJ159945
	R: CGCCTGAAACTTCCACCA					
E7	F: GCGGCCTCCTAGTCACATT	(TCT)_9_	53	283–291	3	KJ159947
	R: CACCACCTTTGCCTTCCTT					
E10	F: CACCGTCACTCCTAACCA	(TC)_9_	50	193–205	4	KJ159951
	R: ATGCAGGAAAGGAAGTCG					
Z3	F: GTCGGAGAACTATCGAGAT	(CT)_11_	53	123–135	3	KJ159953
	R: GAGGAAGAGCGTGGAGGA					
Z5	F: ATATGTTTTACTTGGTGG	(AG)_13_	50	190–201	5	KJ159957
	R: CTTCCTCTTATTTGCTAG					
Z6	F: TCCCAATTTGCACTTTCA	(CTT)_9_	50	174–195	4	KJ159955
	R: ACCCACCAACAACATAAGC					
Z11	F: GGTTGTATGCCATCGTCG	(AG)_3_AA(AG)_6_	50	201–208	2	KJ159952
	R: CCTTTCTGCCGTGATTTT					
Z12	F: ATTGTTTTCATCGCTCTA	(TC)_10_	50	190–201	9	KJ159949
	R: GGAGAAAGGTTGATAGGAG					
Z14	F: CAGGTGGTGGGCTGGTAAT	(GA)_6_	56	170–180	3	KJ159941
	R: CCTCGGTTCCGCCATTTGT					
Z16	F: CCCTCGGTTCCGCCATTT	(GT)_7_	52	155–170	2	KJ159937
	R: GGTGGTGGGCTGGTAATG					

Note

*A* = number of alleles; *T*_a_ = annealing temperature.

Numbers of alleles per locus, observed heterozygosity (*H*_o_), and expected heterozygosity (*H*_e_) were calculated by GenAlEx 6.5 (Peakall and Smouse, 2012); linkage disequilibrium and deviations from Hardy–Weinberg equilibrium were estimated using GENEPOP version 3.4 (Rousset, 2008). Only 11 primer pairs displayed polymorphism among these three populations (Table 1), and the amplification products were within the expected size range. The number of alleles per locus ranged from two to nine, with an average of 3.81 alleles. The average levels of *H*_o_ and *H*_e_ in all three populations were 0.31 ± 0.06 and 0.29 ± 0.04, respectively (Table 2). Five loci (E10, Z6, Z11, Z14, Z16) in the Lijiang population, three loci (Z5, Z11, Z14) in the Yanyuan population, and four loci (E7, E10, Z11, Z12) in the Weining population showed significant deviations from Hardy–Weinberg equilibrium, indicating heterozygote deficiencies. In addition to the 11 polymorphic loci, 10 monomorphic microsatellite loci were obtained and the sequence information was deposited to NCBI (Appendix 2).

**Table 2:** Genetic properties of 11 newly developed polymorphic microsatellite markers for *Psammosilene tunicoides*.^a^

Locus	Lijiang population (*n* = 18)			Yanyuan population (*n* = 20)			Weining population (*n* = 20)		
	*A*	*H*_o_	*H*_e_ b	*A*	*H*_o_	*H*_e_ b	*A*	*H*_o_	*H*_e_ b
E2	2	0.200	0.180	2	0.100	0.095	2	0.200	0.180
E5	3	0.200	0.185	1	0.000	0.000	3	0.500	0.395
E7	2	0.400	0.420	1	0.000	0.000	2	0.000	0.420^**^
E10	3	1.000	0.545^**^	4	1.000	0.615	3	1.000	0.545^**^
Z3	1	0.000	0.000	2	0.100	0.095	2	0.100	0.095
Z5	1	0.000	0.000	3	0.100	0.485^**^	2	0.100	0.095
Z6	3	0.100	0.185^***^	2	0.100	0.095	3	0.300	0.615
Z11	2	0.900	0.495^**^	2	1.000	0.500^**^	2	1.000	0.500^**^
Z12	5	0.600	0.720	3	0.400	0.595	4	0.600	0.595^**^
Z14	2	0.000	0.180^**^	2	0.000	0.180^**^	2	0.100	0.095
Z16	2	0.000	0.180^**^	2	0.100	0.255	2	0.100	0.095

Note

*A* = number of alleles; *H*_e_ = expected heterozygosity; *H*_o_ = observed heterozygosity; *n* = number of individuals.

a Locality and voucher information are provided in Appendix 1.

b Significant deviation from Hardy–Weinberg equilibrium (*P < 0.01, **P < 0.05, ***P < 0.001).

We also tested 11 primer pairs in three species of the related genus *Silene*:* S. gracilicaulis*,* S. huguettiae*, and *S. gonosperma*. The results revealed that only three primers (E5, Z3, and Z14) show amplified bands, with lower *H*_o_ and *H*_e_ in these loci (Table 3).

**Table 3:** Cross‐amplification of microsatellite loci developed for *Psammosilene tunicoides* in three related *Silene* species.^a^

Locus	*Silene gracilicaulis* (*n* = 6)				*Silene huguettiae* (*n* = 5)				*Silene gonosperma* (*n* = 6)			
	*A*	*H*_o_	*H*_e_	Allele size range (bp)	*A*	*H*_o_	*H*_e_	Allele size range (bp)	*A*	*H*_o_	*H*_e_	Allele size range (bp)
E2	—	—	—	—	—	—	—	—	—	—	—	—
E5	4	0.667	0.681	193–209	1	0.000	0.000	201–231	1	0.000	0.000	183–209
E7	—	—	—	—	—	—	—	—	—	—	—	—
E10	—	—	—	—	—	—	—	—	—	—	—	—
Z3	1	0.000	0.000	225	1	0.000	0.000	225	2	0.200	0.180	225
Z5	—	—	—	—	—	—	—	—	—	—	—	—
Z6	—	—	—	—	—	—	—	—	—	—	—	—
Z11	—	—	—	—	—	—	—	—	—	—	—	—
Z12	—	—	—	—	—	—	—	—	—	—	—	—
Z14	1	0.000	0.000	201	1	0.000	0.000	201–217	2	0.333	0.278	201–217
Z16	—	—	—	—	—	—	—	—	—	—	—	—

Note

— = unsuccessful amplification; *A* = number of alleles; *H*_e_ = expected heterozygosity; *H*_o_ = observed heterozygosity; *n* = number of individuals.

a Locality and voucher information are provided in Appendix 1.

## CONCLUSIONS

This is the first study to characterize microsatellite markers specifically for *P. tunicoides*. The 11 polymorphic markers developed here will enable further studies investigating the population genetic structure, the development of conservation strategies, and marker‐assisted selection breeding of this species. However, the cross‐species amplification of these markers indicates that they may be less useful in related genera, such as *Silene*, because of the distant phylogenetic relationships between *P. tunicoides* and other species in Caryophyllaceae.

## DATA ACCESSIBILITY

Sequence information for the developed primers has been deposited to the National Center for Biotechnology Information (NCBI); GenBank accession numbers are provided in Table 1.

## APPENDIX 1 Voucher and locality information of three populations of *Psammosilene tunicoides* and three *Silene* species used in the study.^a^

**Table:**

Taxon	Population code	Voucher specimen accession no.^b^	*N*	Locality	Geographic coordinates	Altitude (m)
*Psammosilene tunicoides* W. C. Wu & C. Y. Wu	LJ	LGD2016005	18	Lijiang, Yunnan Province, China	26°16′06″N, 100°16′16″E	2381
*P. tunicoides*	YY	LGD2016018	20	Yanyuan, Sichuan Province, China	27°06′37″N, 100°42′09″E	2650
*P. tunicoides*	WN	LGD2016020	20	Weining, Guizhou Province, China	27°06′58″N, 104°07′27″E	2400
*Silene gracilicaulis* C. L. Tang	XGLL	MS2017230	6	Xianggelila, Yunnan Province, China	27°32′18″N, 99°43′08″E	3240
*S. huguettiae* Bocquet	XJ	MS2017506	5	Xiaojin, Sichuan Province, China	30°54′42″N, 102°53′49″E	5040
*S. gonosperma* (Rupr.) Bocquet	JL	MS2017536	6	Jiulong, Sichuan Province, China	28°22′39″N, 101°37′32″E	2135

Notes

*N* = sample size for each population.

a Vouchers are stored in the herbarium of Yunnan University of Traditional Chinese Medicine, Kunming, Yunnan, China.

b LGD = Guodong Li; MS = Xiangguang Ma and Wenguang Sun.

## APPENDIX 2 Characteristics of 10 monomorphic microsatellite loci developed in *Psammosilene tunicoides*.

**Table:**

Locus	Primer sequences (5′–3′)	Repeat motif	*T*_a_ (°C)	Allele size (bp)	GenBank accession no.
E1	F: CCCTTAGTTGTTACTTTCTC	(CA)_3_A(CA)_5_	50	230	KJ159936
	R: TTGATTACTTCTTCGCCAC				
E3	F: ACTTCGAGCAGAACAGACT	(CA)_6_	50	122	KJ159939
	R: CAAATGGGACACTATAAATG				
E4	F: TTTCTATCCAAAAGGCACT	(CT)_4_TT(CT)_6_	48	221	KJ159941
	R: CAAACATAAGCAACATTCA				
E6	F: TGGTCAAAGTAGGCAACA	(AG)_8_	52	117	KJ159942
	R: CCACGTACCCAATCAAAT				
E8	F: GCCATTGATTACTTCTTCG	(GT)_5_T(GT)_3_	56	236	KJ159943
	R: AGCCCTTAGTTGTTACTTTCTC				
E9	F: AACGCAACGCAGTCCCTC	(TC)_6_	52	222	KJ159944
	R: ACCCAAGAATCCGTCCTA				
E11	F: CCACGTACCCAATCAAATA	(CT)_7_	50	147	KJ159946
	R: TGGTCAAAGTAGGCAACAC				
E12	F: GAGAATTGGAGGGTGTAG	(GT)_5_	48	147	KJ159948
	R: ACCTGAGAAAGATGGGAC				
Z1	F: GCCATTGATTACTTCTTCG	(TC)_6_	53	192	KJ159950
	R: AGCCCTTAGTTGTTACTTTCTC				
Z2	F: TCAATGCAATTTAGGAGGAA	(GA)_4_	50	238	KJ159954
	R: TGCTTGTTGAACCCTGTG				

Note

*T*_a_ = annealing temperature.
